# Appendiceal inversion secondary to endometriosis and dilated mucinous adenoma identified on screening colonoscopy

**DOI:** 10.1093/jscr/rjag291

**Published:** 2026-04-23

**Authors:** Rhianna Bhatia, David T Guerrero, Neetu Chahil, Hongfa Zhu, Asha G Bale

**Affiliations:** Hackensack Meridian School of Medicine at Hackensack University Medical Center, 123 Metro Blvd, Nutley, New Jersey 07110, United States; Department of Surgery, Hackensack University Medical Center, 30 Prospect Ave, Hackensack, New Jersey 07601,United States; Department of Gastroenterology, Hackensack University Medical Center, 30 Prospect Ave, Hackensack, New Jersey 07601, United States; Department of Pathology, Hackensack University Medical Center, 30 Prospect Ave, Hackensack, New Jersey 07601, United States; Department of Surgery, Hackensack University Medical Center, 30 Prospect Ave, Hackensack, New Jersey 07601,United States

**Keywords:** appendix intussusception, endometriosis, mucinous adenoma, colonoscopy, appendectomy, dual pathology

## Abstract

Appendix intussusception (AI), with ~200 reported cases, is a rare condition in which the appendix invaginates into the cecum [1]. Etiologies include benign and malignant pathologies including both intraluminal and extraluminal lesions, but dual pathology has not been described previously. We present a case of a 52-year-old female with colonoscopic findings suspicious for AI or a submucosal lesion. Surgical pathology following appendectomy revealed AI caused by both endometriosis and a mucinous adenoma. AI is usually diagnosed incidentally on laparoscopy. Though rarely diagnosed preoperatively, recognizing AI on colonoscopy is critical. To our knowledge, this is the first reported case of AI due to both endometriosis and mucinous neoplasm.

## Introduction

Appendix intussusception (AI) occurs when the appendix invaginates into the lumen on the cecum [2]. In the absence of a prior open appendectomy and concurrent iatrogenic cause, AI is exceedingly rare, representing an incidence of 0.01% [3]. Fewer than 10 published cases describe preoperative diagnosis on colonoscopy or Computed tomography (CT) scan [3, 4]. This report presents a case of an otherwise healthy female in whom AI was identified during her first screening colonoscopy.

## Case report

A 52-year-old female presented for her first screening colonoscopy. She denied abdominal or pelvic pain, constipation, diarrhea, or melena. The patient denied any weight loss, fatigue, fevers, or chills. Her medical history included asthma and chronic migraine headaches. Surgical history was notable for a cesarean section, tonsillectomy, and an endometrial ablation for unknown indication 10 years prior. Family history was negative for colorectal cancer or other malignancies, and she had no history of smoking.

On physical examination, the patient was afebrile, with a blood pressure of 129/86, pulse of 88 bpm, and a BMI of 20.92 kg/m^2^. The examination showed no abdominal tenderness or distention. A preoperative chest X-ray showed normal lungs.

**Figure 1:**
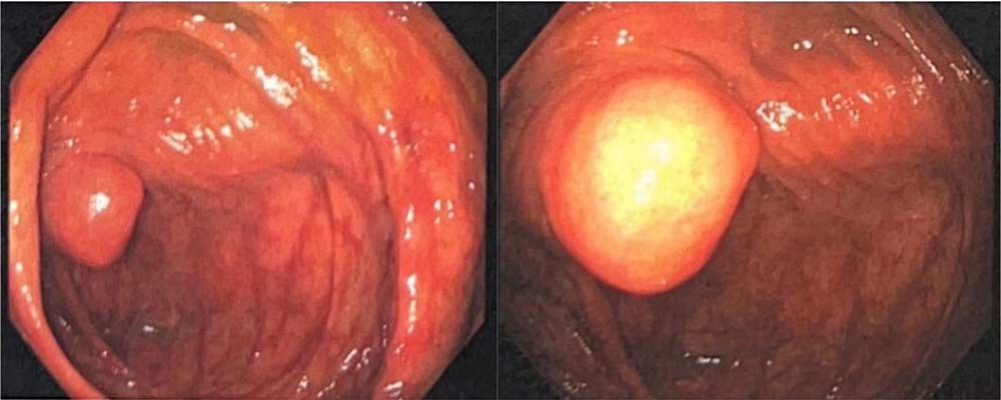
Colonoscopic findings demonstrating AI the appendiceal orifice is not visualized, revealing a smooth, round mass protruding into the cecum. Further advancement of the scope shows the inverted appendiceal tip within the cecal lumen.

Colonoscopy revealed four benign polyps, including three tubular adenomas located in the cecum, ascending colon, and hepatic flexure, and a 3 mm area of hyperemia in the hepatic flexure. A 2 cm cecal submucosal lesion was biopsied, showing mild focal colitis. Additionally, an incidental 2 cm appendiceal lesion, suspicious for a prolapsed appendix or submucosal mass, was identified, prompting a referral to general surgery for appendectomy (Fig. 1).

Preoperative computerized tomography enterography was normal and showed no intraluminal masses or signs of inflammatory change in the large or small bowel. Additionally, the liver, gallbladder, pancreas, uterus, and appendix and adnexa appeared unremarkable with no identifiable masses. Preoperative laboratory results were within normal limits, including a white blood cell count of 7.8/μl (RR: 4.0–11.0 × 10^3^/μl), hemoglobin count of 13.3 g/dl (RR: 12.0–15.5 g/dl), platelets of 299/μl (RR: 135–430 × 10^3^/μl), and normal electrolytes.

### Treatment

The patient underwent elective laparoscopic appendectomy, which was converted to open procedure to better isolate the base of the appendix. On laparoscopy, there were no mucinous ascites, peritoneal implants, or endometriosis seen grossly in the abdomen or pelvis. The appendix was found to be telescoped and firm. The mesoappendix was isolated and divided with a 45 mm stapler. The cecum, ileum, and appendiceal stump were exteriorized through the umbilical incision, confirming the diagnosis of inverted appendix. A 45 mm linear stapler was fired across the cecum, to resect both the extra- and intraluminal appendix. The remainder of the postoperative course was uncomplicated, with discharge home the same day.

### Outcome and follow-up

**Figure 2:**
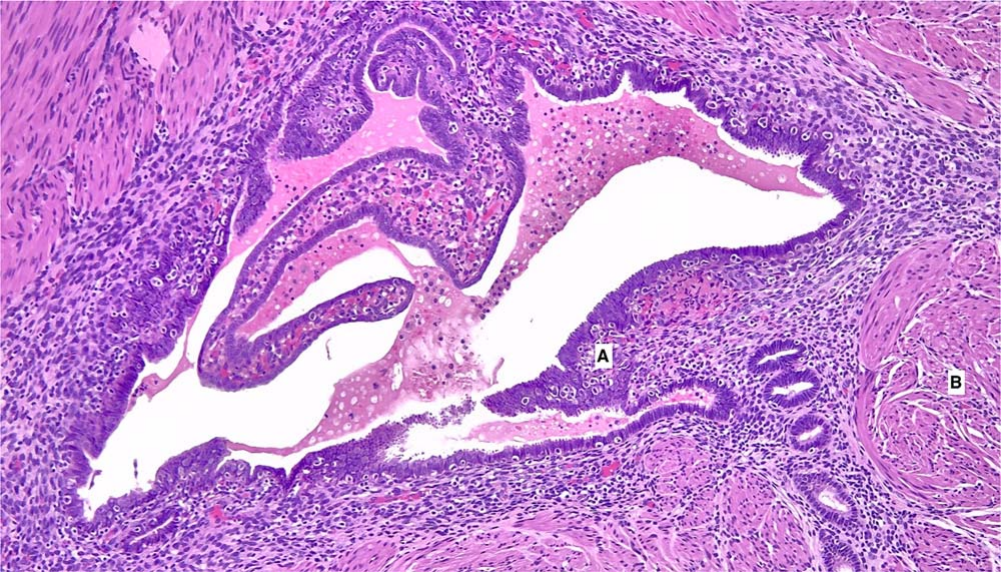
Appendiceal wall with endometriosis (100x magnification). A. Endometrial gland and stromal cells embedded within the muscularis propria of the appendix. B. Surrounding smooth muscle stroma indicative of fibromuscular hypertrophy.

**Figure 3:**
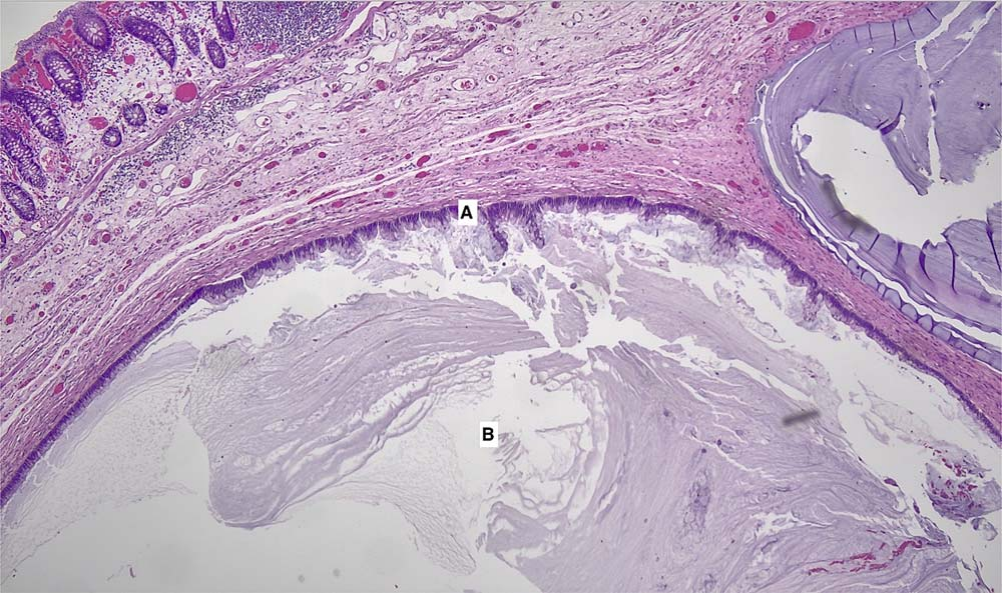
Appendix with low-grade appendiceal neoplasm (LAMN) (40x magnification). A. Vilous proliferation of mucin-producing cells with minimal nuclear atypia with “pushing” invasion into the wall. B. Dilated space with abundant mucin indicating core of mucinous adenoma.

Final surgical pathology revealed an inverted appendix with endometriosis and prominent surrounding fibromuscular hypertrophy, which was the cause of obstruction of the appendiceal lumen (Fig. 2). Additionally, the adjacent cecal colonic submucosa showed dilated mucinous space lined by epithelium with low-grade dysplasia, consistent with mucinous adenoma (Fig. 3). Margins were negative, and no mucin material was found on the appendiceal or cecal serosal surface, indicating an intact capsule and no mucinous spill into the peritoneal cavity.

The patient recovered without complications postoperatively. The patient was referred to medical oncology for surveillance of low-grade appendiceal mucinous neoplasm, repeat colonoscopy in one year, and referral to gynecology for the new diagnosis of endometriosis.

## Discussion

We present a case of AI diagnosed on the first screening colonoscopy of an otherwise healthy female, whose only symptom was occasional hematochezia. Final pathology revealed that the AI was due to both endometriosis with prominent fibromuscular hypertrophy and dilated mucinous adenoma. While previous reports have described AI caused solely by endometriosis or low-grade mucinous neoplasms, this is the first report to identify both pathologies as its cause [5, 6].

**Figure 4:**
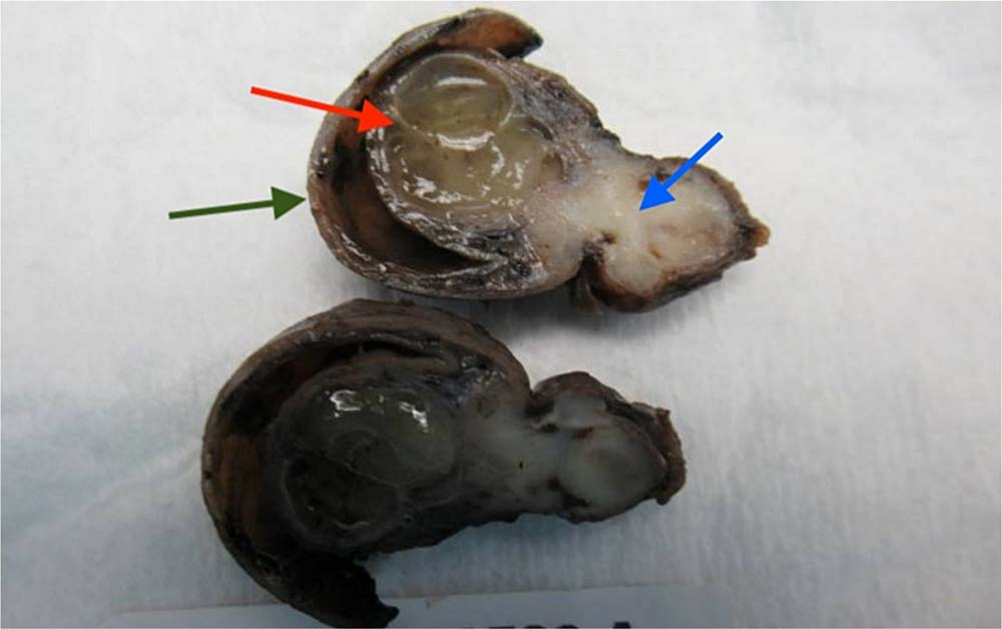
Gross specimen from appendectomy with partial cecectomy, appendix inverted into the cecum (green) with dilated mucinous adenoma (red) and endometriosis with fibrosis obstructing the appendiceal lumen (blue).

The pathophysiology for AI is not fully understood due to its rarity; however, it is theorized that local irritation and inflammation lead to abnormal peristalsis, resulting in the invagination of the appendix into itself or the cecum. In this case, we propose that fibromuscular hypertrophy from long-standing appendiceal endometriosis caused complete appendiceal luminal obstruction. Unlike typical mucinous neoplasms, which slowly dilate the appendix and produce indolent symptoms, this obstruction led to inversion of the mucinous adenoma into the cecum, resulting in earlier detection on endoscopy (Fig. 4).

Symptoms of AI can be non-specific, including intermittent right lower abdominal pain, changes in bowel habits, hematochezia, melena, and intestinal obstruction, with some cases presenting without symptoms. AI may be misidentified as a polyp or cecal mass on colonoscopy or CT scan. Etiologies include foreign bodies, fecaliths, mucoceles, Crohn’s disease, lymphoid hyperplasia, and polyps. Carcinoid or other malignant neoplasm can be the cause of AI as well, and therefore, whenever AI is diagnosed or suspected, surgery is mandated. Given the colonoscopic findings suggestive of AI or a submucosal lesion, appendectomy was advised to further evaluate the abnormality, mitigate potential complications, and exclude malignancy. Appendiceal endometriosis, although rare, is a recognized benign cause of an inverted appendix, with fewer than 30 cases in the literature [7].

Recognizing AI on colonoscopy is crucial to prevent endoscopic removal of misdiagnosed polyps or cecal masses, which can result in pseudomyxoma peritonei (PMP) in the case of an underlying appendiceal mucinous neoplasm. Furthermore, final pathology after appendectomy with partial cecectomy can inform the management of the underlying cause [8, 9]. Both endometriosis and mucinous adenomas are benign conditions that do not require further management. On the contrary, further workup and/or prophylactic hemicolectomy may be necessary after diagnosis of malignant etiologies of AI.

## Conclusion

This case illustrates that AI can be the initial presentation of asymptomatic endometriosis or appendiceal mucinous adenoma. While most cases are diagnosed incidentally during appendectomy, rare instances can be identified on colonoscopy, as demonstrated here in the first reported case of dual underlying pathology. Surgical management, including appendectomy and partial cecectomy, is warranted. Final histopathology will guide subsequent treatment and surveillance.
